# A Comprehensive Examination of Percutaneous Endoscopic Gastrostomy and Its Association with Amyotrophic Lateral Sclerosis Patient Outcomes

**DOI:** 10.3390/brainsci9090223

**Published:** 2019-09-04

**Authors:** Leila Bond, Paulamy Ganguly, Nishad Khamankar, Nolan Mallet, Gloria Bowen, Braden Green, Cassie S. Mitchell

**Affiliations:** Laboratory for Pathology Dynamics, Department of Biomedical Engineering, Georgia Institute of Technology and Emory University School of Medicine, Atlanta, GA 30332, USA

**Keywords:** ALS, motor neuron disease, dysphagia, malnutrition

## Abstract

There is literature discord regarding the impact of percutaneous endoscopic gastrostomy (PEG), or “feeding tube”, on amyotrophic lateral sclerosis (ALS) outcomes. We assess one of the largest retrospective ALS cohorts to date (278 PEG users, 679 non-users). Kruskal–Wallis and Kaplan–Meier analysis compared cohort medians and survival duration trends. A meta-analysis determined the aggregate associative effect of PEG on survival duration by combining primary results with 7 published studies. Primary results (*p* < 0.001) and meta-analysis (*p* < 0.05) showed PEG usage is associated with an overall significant increase in ALS survival duration, regardless of onset type. Percent predicted forced vital capacity (FVC %predict) ≥50 at PEG insertion significantly increases survival duration (*p* < 0.001); FVC %predict ≥60 has the largest associative benefit (+6.7 months, *p* < 0.05). Time elapsed from ALS onset until PEG placement is not predictive (*p* > 0.05). ALSFRS-R survey assessment illustrates PEG usage does not slow functional ALS pathology (*p* > 0.05), but does stabilize weight and/or body mass index (BMI) (*p* < 0.05). Observed clinical impression of mood (CIM), was not impacted by PEG usage (*p* > 0.05). Overall results support PEG as a palliative intervention for ALS patients with ≥50 FVC %predict at PEG insertion.

## 1. Introduction

Amyotrophic lateral sclerosis (ALS) is a progressive neurodegenerative disease that damages motor neurons. Patients eventually develop dysphagia (difficulty swallowing), leading to weight loss or even life-threatening asphyxiation. A common intervention to assist with nutrition and hydration for dysphagic ALS patients experiencing rapid weight decline [1] is a percutaneous endoscopic gastrostomy (PEG)—a procedure where a “feeding tube” is inserted through the abdominal wall directly into the stomach [2,3,4]. PEG slows rapid weight loss and improves patient nutrition [5]. PEG typically delivers 30% of the patients’ daily caloric intake [6]. Prevention of rapid weight decline [7] and having a higher pre-morbid body mass index (BMI) [8,9] is thought to positively contribute to survival [10,11]. Naturally, a physician’s choice to prescribe PEG and a patient’s choice to accept it must take into account the heterogeneity of ALS, symptoms most troubling to each individual patient, the patient’s age of onset, and the patient’s personal preferences about surgery and supplementary nutrition. This study focuses on the more quantifiable aspects of PEG and its prescription, such as percent predicted forced vital capacity (FVC %predict), a measure of respiratory function. In the United States, the standard clinical protocol is for a dysphagic ALS patient or an ALS patient with more than 10% premorbid weight loss to receive a PEG while FVC %predict is ≥50 [12], although some studies have shown the cutoff to be lower [13]. While PEG is a relatively common ALS intervention, there is disagreement in the literature regarding the positive association of PEG usage with increased survival duration. The present study aims to quantitatively analyze the effect of PEG usage on ALS functional progression, quality of life, and associative benefit to survival disease duration. Important parameters used to analyze the impact of PEG usage include: disease duration, FVC %predict [14], Amyotrophic Lateral Sclerosis Functional Rating Scale Revised (ALSFRS-R) score [15,16,17,18], clinical impression of mood (CIM) [19], body weight, and body mass index (BMI) [20]. Assessment of statistical relationships between PEG usage and the aforementioned parameters is performed using one of the largest ALS PEG user study cohort sizes to date. Finally, a meta-analysis that combines present study results with the results of previous ALS PEG studies is performed to determine an overall, aggregate trend that can provide data-enabled evidence and clarity for clinical decision support of PEG in ALS.

## 2. Materials and Methods

This is a retrospective analysis of 8028 clinical visit records collected from 1585 patients at the Emory ALS Clinic (Emory University Hospital, Atlanta, GA, USA). Data collection, organization, and quality control methods are as previously published in prior work with this data set [21,22,23,24,25,26]. All data was de-identified and anonymized. Institutional Review Board approval was obtained from Georgia Institute of Technology and Emory University. For the present retrospective study, primary data fields for each patient visit include: onset type, PEG placement date, PEG usage (e.g., calories/day), body weight, body mass index (BMI), combined or total score for ALSFRS-R survey [15,16], and FVC %predict. Clinical impression of mood (CIM) [19], patient gait score, and temporal progression was calculated for each patient visit using methods briefly outlined below and described in more detail in the Supplement (Supplementary Tables S1 and S2). ALS onset type was defined according to previous standardized definitions [23] for limb onset, bulbar onset, and “unclassifiable” onset. 

### 2.1. Study Inclusion Criteria

Only patients with an explicit date of death and with complete information on PEG usage (denoted throughout as “PEG users”, or U for “used”) or PEG disuse (denoted throughout as “non-users”, or DNU for “did not use”) were included. Table 1 illustrates included sample sizes categorized by PEG usage (users, non-users) and ALS onset type (limb, bulbar, unclassifiable). Included PEG users verifiably utilized PEG from the date of insertion until death, whereas PEG non-users verifiably never had a PEG inserted.

### 2.2. Calculation of Survival Duration

For the primary study results, survival duration calculation is performed using the first ALS clinic visit date and the official recorded date of death; “first clinic visit date” was chosen instead of recorded “date of first symptom onset” (the latter is a field that is entered according to patient memory). First clinic visit was chosen to ensure data completeness and veracity, and it is the preferred method based on recent assessments of the accuracy of ALS survival duration [23,26]. However, for the meta-analysis results, survival duration was calculated using the patient-reported date of first symptom onset and the official recorded date of death to ensure comparability between studies.

### 2.3. Temporal Disease Assessments

In addition to standard survival duration, two different temporal disease assessment measure methods, “thirds” and “quartiles”, were used to assess how the timing of PEG placement may be associated with changes in functional metrics or survival duration. For the thirds method, each PEG user’s disease duration was divided into trimesters; each PEG user was then classified by the trimester in which their first PEG was placed (e.g., first, second, or third trimester). Similarly, for the quartiles method, each PEG user was classified by the fraction of the disease duration elapsed (in quartiles) at the time their first PEG was placed (e.g., first, second, third, fourth quartile).

### 2.4. Clinical Impression of Mood

For each clinic visit, the clinician denoted a short, qualitative description of the patient’s exuded mood [19]. Text mining of the clinic notes was used to classify clinical impression of mood (CIM) using a binomial score of 0 or 1. A perceived positive or neutral mood for the visit day was assigned a “0”, whereas a perceived negative mood was given a value of “1”. Details of the text mining CIM scoring system [19] are shown in Supplementary Table S1. Changes of visit-specific CIM for each patient were analyzed for PEG users and PEG non-users. Note that CIM scores for patients with documented pseudobulbar affect were excluded for this portion of the study to ensure integrity of results [19], given the exuded emotion and facial expression of a patient with pseudobulbar affect would not necessarily match their actual visit-specific mood. CIM was utilized due to a lack of standardized quality of life (QoL) surveys for this data set. The advantage of CIM is the lack of patient or caretaker survey bias [19,27]. The limitation of CIM is the reliance on clinician observed interpretation of verbalized communication or non-verbalized facial expression as an assessment of patient mood.

### 2.5. Gait

Gait score is used as an additional or adjunctive metric of ALS functional progression beyond the patient-driven ALSFRSR survey score [15,16]. For each clinic visit date, the clinician denoted a short, qualitative description of the patient’s visualized gait or walking ability. Text mining of visualized patient gait description was utilized to assign a patient visit gait score ranging from 1–8 as shown in Supplementary Table S2. A value of 1 indicated able, independent walking, while higher numbers indicated more impairment, such as spasticity, or whether the patient used an assistive device such as a walker, cane, or brace. Changes between visit-specific scores for each patient were analyzed for PEG users and non-users.

### 2.6. Statistical Analysis of Primary Results

Data distributions for the PEG users and non-users were evaluated separately using the Shapiro–Wilk test to assess data normality. Shapiro–Wilk results illustrated that the data was non-normal. Thus, statistical analysis to compare medians was performed using the Kruskalz–Wallis test, a non-parametric test of variance between cohorts. Assessment includes comparing median and interquartile range. Significance was set to be *p* < 0.05, with distinctions in significance noted as *p* < 0.05 or *p* < 0.001.

### 2.7. Literature Meta-Analysis of PEG Usage in ALS

A meta-analysis was performed to examine the aggregate results of a multitude of published studies that examined PEG usage in ALS patients. A search in PubMed was performed using keywords “PEG and “ALS” (including synonyms) to identify studies that measured survival duration in PEG users and PEG non-users. Selected papers included patient group sample sizes for PEG users and non-users and quantitative *p*-values for significant change in ALS survival duration. Seven studies were identified for inclusion in the meta-analysis. The *p*-values from these published studies were used to determine the chi squared value via Fisher’s method. The aggregate p-value associated with the resultant chi squared value was found in MATLAB (The Mathworks, Inc., Natick, MA, USA) using a degree of freedom of 4 and significance level of 0.05. 

## 3. Results

As shown in Table 1, the study included 278 PEG users and 649 PEG non-users; distribution of onset type matched that of the general ALS population. The key assessment was to determine the association of PEG usage with ALS survival duration. As noted in the methods, the primary study results report survival duration using the first ALS clinic visit and the recorded of date of death, as this definition has been found to increase results integrity [23,28]. Other parameters examined include: ALS onset type, FVC %predict, change in total ALSFRS-R score, change in gait score, and change in clinical impression of mood (CIM). Finally, a statistical meta-analysis was performed to compare the present study’s results and to calculate the combined or aggregate effect of PEG usage on ALS survival duration. The statistical meta-analysis utilized the traditional survival duration definition (time of patient-reported first symptom onset to recorded date of death) to ensure equivalence between included studies.

### 3.1. Assessment of Survival Duration

**Figure 1:**
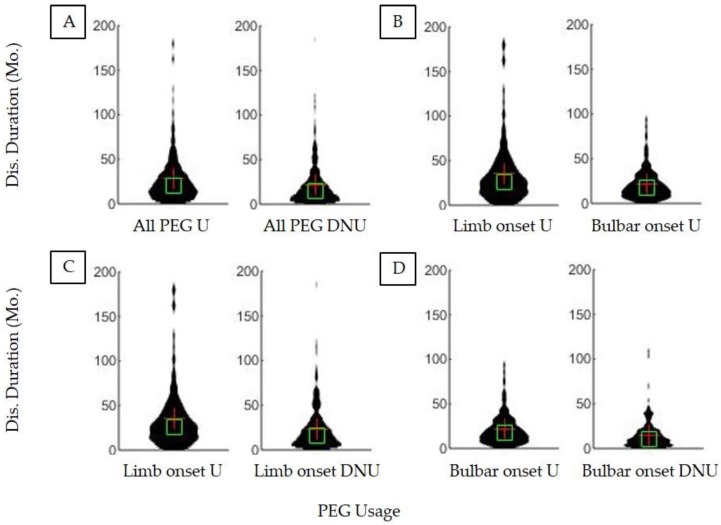
Differences in ALS survival duration based on ALS onset type and PEG usage. (**A**) All PEG users (*n* = 279) had a significantly (*p* < 0.05) longer disease duration than all non-users (*n* = 649). (**B**) Limb onset PEG users (*n* = 138) had significantly longer disease durations than bulbar onset PEG users (*n* = 133) (*p* < 0.05). (**C**) PEG is associated with a significant increase in survival duration in limb onset PEG users (*n* = 138) compared to limb onset non-users (*n* = 448) (*p* < 0.05). (**D**) PEG usage is associated with a significant increase in survival duration in bulbar onset PEG users (*n* = 133) compared to bulbar onset non-users (*n* = 164) (*p* < 0.05).

Figure 1 illustrates ALS patient survival duration on the basis of PEG usage as well as ALS onset type. The median survival duration (defined as time elapsed from first ALS clinic visit until recorded date of death) for all ALS PEG users was 20.8 months (IQR = 23.6) versus 14.8 months for all non-users (IQR = 20.2). Thus, there is a significant (*p* < 0.001) associative survival benefit of ALS PEG usage of +6.1 months (Figure 1). Limb onset PEG users survived 25.9 months (IQR = 29.4) whereas limb onset non-users survived 16.2 months (IQR = 22.4); thus, limb onset PEG usage associative survival benefit is +9.7 months. Bulbar onset PEG users survived 17.1 months (IQR = 19.9) whereas bulbar non-users survived 10.6 months (IQR = 14.9); thus, bulbar onset PEG associative survival benefit was +6.5 months. The positive associative survival benefit of PEG usage is significant for both onset types (*p* < 0.001). 

As noted in the methods, to increase analytical integrity [23,26], the primary results of the present study calculate and report survival duration as time elapsed since the first ALS clinic visit date [when a formal ALS diagnosis was made] until the recorded date of death. Diagnostic delay (time elapsed from patient-reported memory of first symptom until first ALS clinic visit for formal diagnosis) was 10 months for this particular cohort. Thus, for the sake of comparison, 10 months can be added to the above reported measures to convert or approximate “traditional” survival duration (defined as time elapsed since patient-reported memory of first symptom until recorded time of death). 

### 3.2. Assessment of FVC %Predict at PEG Placement

**Figure 2:**
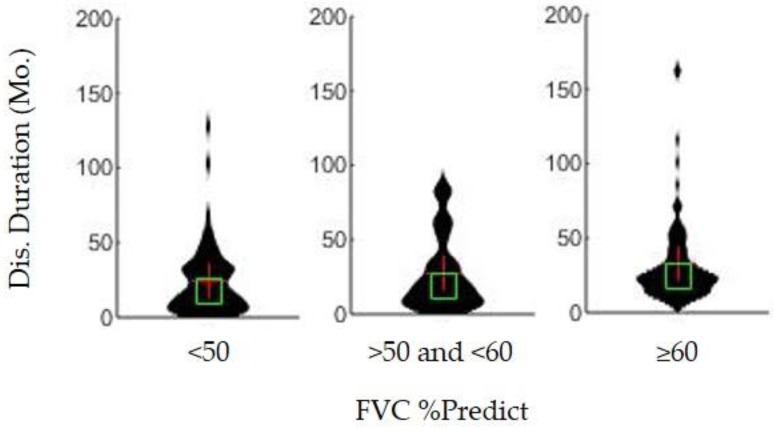
Association of percent of predicted forced vital capacity (e.g., FVC %predict) at the time of PEG placement date on ALS survival duration (in months). Patients with a percent predict of ≥60% (*n* = 95) had significantly higher disease duration than patients with percent predict of <50% (*n* = 57) (*p* < 0.05). Patients with a percent predict between 50 and 60 did not have a significantly different disease duration than the <50% group or the ≥60% group (*p* > 0.05 in both cases).

Current clinical practice typically recommends placement of PEG while percent predicted forced vital capacity (FVC %predict) is >50. As expected, PEG users in the present cohort with FVC %predict ≥50 did have a significantly longer survival duration than PEG users with an FVC %predict <50 at the time of PEG placement (*p* < 0.001). However, the associative benefit is even more pronounced among PEG users who had an FVC %predict ≥60 at the time of PEG placement (*p* < 0.05). Survival duration for FVC %predict ≥60 is 24.2 months (IQR = 21.3) whereas FVC %predict is <50 is 17.5 months (IQR = 26.6), as shown in Figure 2. Associative survival benefit for patients with FVC %predict ≥70, ≥80, and ≥90 were also calculated, but the ≥60 group had the largest significant survival increase from the <50 patient group.

### 3.3. Timing of PEG Placement

**Figure 3:**
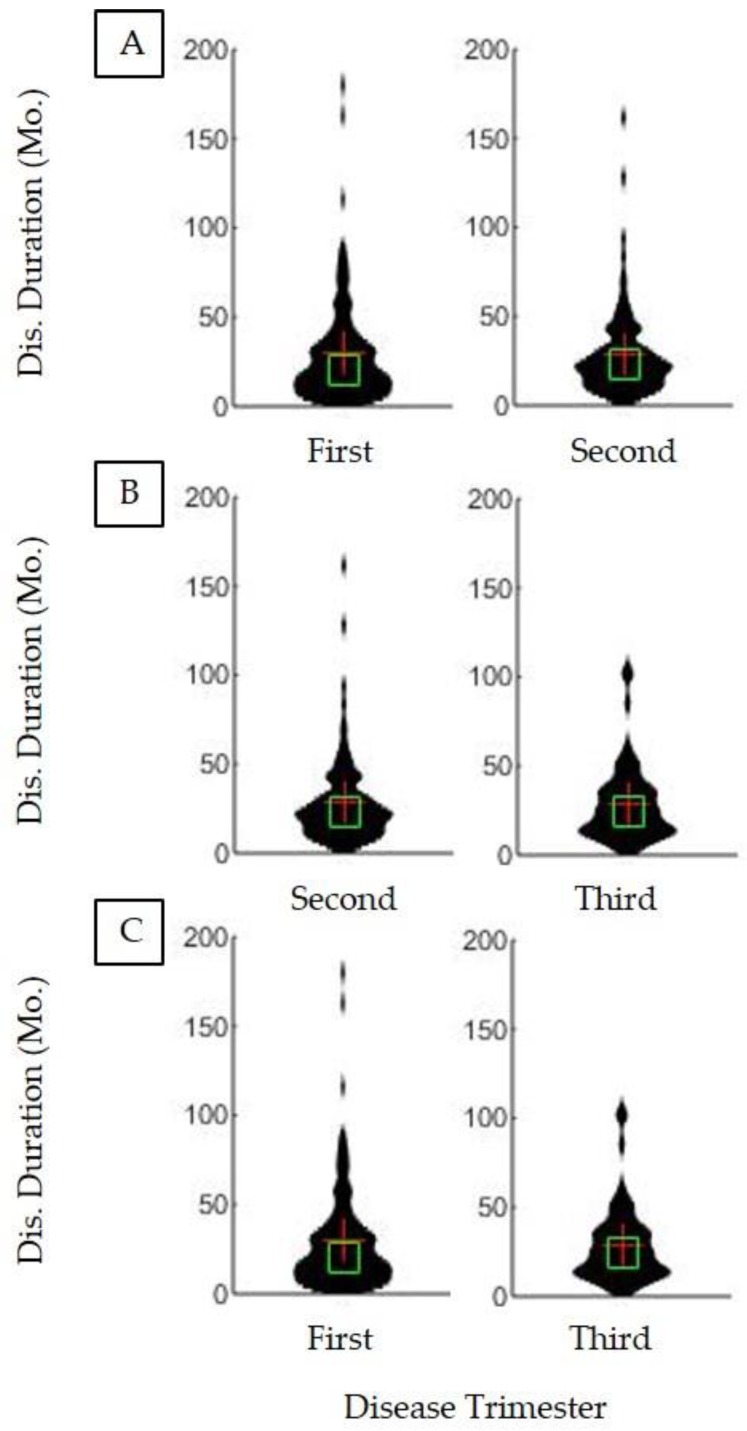
Effect of trimester of disease duration at PEG placement on remaining disease duration. Each of the PEG users’ disease durations was divided into three equal time periods, called trimesters. (**A**) Patients who began PEG in the first trimester (*n* = 116) did not have significantly different disease durations than those who began PEG in the second trimester (*n* = 87) (*p* > 0.05). (**B**) Patients who began PEG in the second trimester (*n* = 87) did not have significantly different disease durations than patients in the third trimester (*n* = 53) (*p* > 0.05). (**C**) Patients who began PEG in the first trimester (*n* = 116) did not have significantly different disease durations than those who began PEG in the third trimester (*n* = 53) (*p* > 0.05).

Figure 3 illustrates that there is no significant survival difference strictly on the basis of when PEG was first placed (*p* > 0.05). While Figure 3 specifically examines the trimester of PEG placement, examination of the impact of PEG placement timing using the quartile method also found no significant impact on survival duration (*p* > 0.5, not shown). Thus, time elapsed since either the first ALS symptom onset or formal ALS diagnosis, alone, is not a meaningful predictor of when PEG should be placed to maximize associative survival benefit.

### 3.4. Impact of PEG on Observed Patient Mood

Examination of the impact on PEG on patient mood is an important indicator of the potential impact of PEG on patient quality of life. Clinical impression of mood (CIM) is a novel binomial categorical metric (e.g., positive mood or negative mood) developed to overcome patient and caretaker survey bias using text mining of electronic medical records; this metric is more specifically defined in Supplementary Table S1. The quartile of PEG placement does not result in a significant difference in the decline of clinical impression of mood (CIM) (*p* > 0.05). The median CIM decline for each quartile was as follows: quartile 1 =0 (IQR = 0.002); quartile 2 = 0.17 (IQR = 0.5); quartile 3 = 0.2 (IQR = 0.5); quartile 4 = 0.25 (IQR = 0.75) as shown in Supplementary Figure S1. Therefore, PEG usage was not associated with significant worsening of clinical impression of mood in PEG users compared to non-users.

### 3.5. Impact of PEG on ALSFRS-R Decline

**Figure 4:**
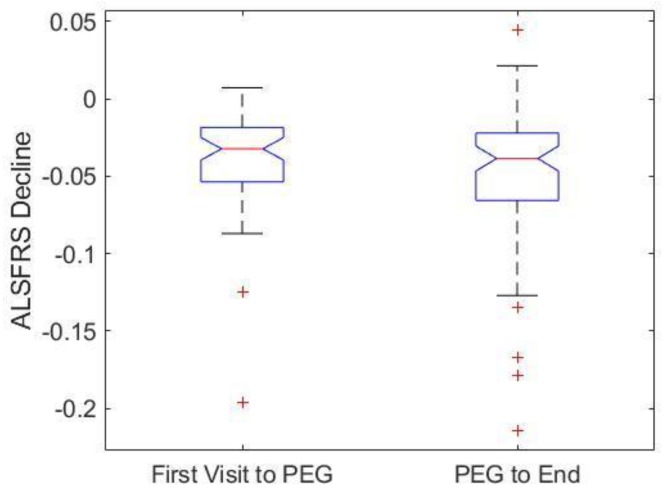
Effect of PEG Placement on change in patient scores for the Amyotrophic Lateral Sclerosis Functional Rating Scale Revised survey (ALSFRS-R). PEG user ALSFRS-R declines were divided between the first ALS clinic visit to PEG placement and from PEG placement to the last recorded clinic visit. ALSFRS-R declines were not significantly different after PEG placement (*p* > 0.05). Thus, PEG placement and usage did not impact rate of ALSFRS-R decline.

The ALSFRS-R score is calculated using a patient survey that assesses activities of daily living and respiratory function [15,16]. ALSFRS-R is currently considered the primary metric for assessing overall ALS disease progression. Decline in ALSFRS-R score signifies disease progression. Notably, PEG placement and usage did not have a significant impact (*p* > 0.05) on the rate of ALSFRS-R score decline (Figure 4). 

Additionally, the potential impact of PEG on ALS function was also assessed using a novel categorical visualized gait score developed using a text mining technique; more specific information on the gait scoring methods is detailed in Supplementary Table S1. Gait scoring analysis reveals no significant changes based on PEG usage (Supplementary Figure S2). These adjunctive gait scoring results re-affirm the ALSFRS-R results, as both illustrate that PEG usage has no impact on ALS patient functional decline, or more specifically, activities of daily living.

### 3.6. Impact of PEG on Body Weight and BMI

Decline in patient body weight and body mass index (BMI) before PEG placement is expected; recall rapid or dramatic weight loss is a primary reason for PEG placement [9]. As expected, PEG users’ percent body weight decline and body mass index (BMI) decline from first ALS clinic visit until PEG placement was significant (*p* < 0.05). However, the percent body weight and BMI decline from PEG placement until last recorded ALS clinic visit was not significant (*p* > 0.05), as shown in Supplementary Figure S3. 

### 3.7. Kaplan–Meier Survival Analysis

**Figure 5:**
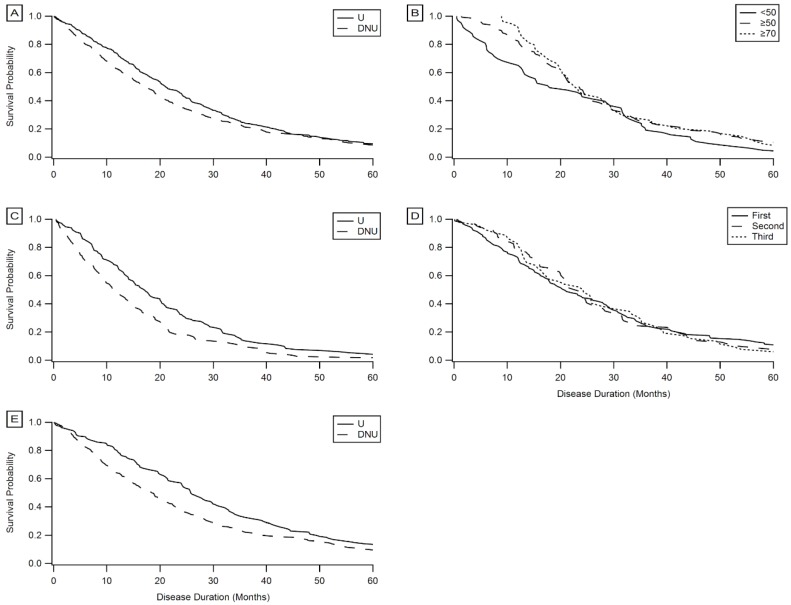
Kaplan–Meier survival curves. (**A**) PEG users (*n* = 279) compared to non-users (*n* = 49). (**B**) PEG users with FVC %predict <50 at initial PEG placement (*n* = 57) compared with FVC %predict ≥50 (*n* = 124) and FVC %predict ≥70 (*n* = 54). (**C**) Bulbar onset PEG users (*n* = 133) compared with bulbar onset non-users (*n* = 64). (**D**) PEG users who began using PEG in the first trimester of disease duration (*n* = 116) compared with those who began in the second (*n* = 87) and third trimesters (*n* = 53). (**E**) Limb onset (*n* = 138) PEG users compared with limb onset non-users (*n* = 448).

In addition to comparison of median survival durations using Kruskal–Wallis, Kaplan–Meier survival probability curves (Figure 5) were constructed to assess temporal trends in survival duration among the different PEG user and non-user populations. These results confirm the results from the above sections: PEG users experience a significant associative benefit in survival duration regardless of onset type (Figure 5A,C,E); time elapsed from onset or first clinic visit until PEG placement is not a predictor of the associative benefit of PEG on survival duration (Figure 5D); and placement of PEG before precipitous respiratory decline results in higher survival probabilities, with the “optimal” FVC %predict at initial PEG placement falling between 50–70 (Figure 5B). 

### 3.8. Meta-Analysis of PEG Cohort Studies

**Figure 6:**
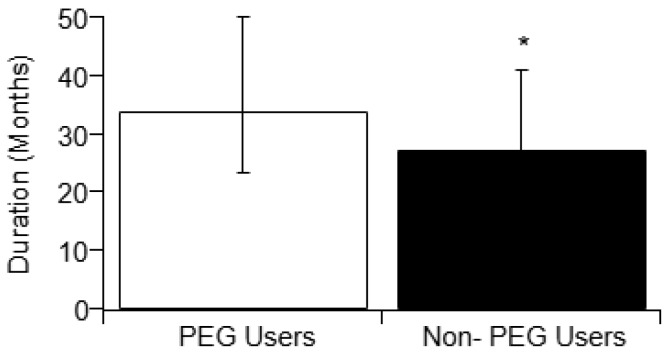
Comparison of median disease durations (with error bars representing interquartile range) of PEG users and non-users from the meta-analysis. Disease durations of PEG users and non-users were recorded from published studies that found either significant or insignificant increase in disease duration correlated with PEG usage (see Table 2). Prior study results were combined with the present study’s results in order to determine the aggregate effect of PEG usage on survival duration. Fisher’s test results (see Methods) illustrate a significant aggregate effect that results in a positive associative survival benefit to ALS PEG users. For consistency, the disease duration for the meta-analysis is defined as time elapsed since patient-reported first symptom onset until recorded date of death.

Prior studies examining PEG usage in ALS have had mixed results. Five prior studies reported an associative qualitative increase in survival duration with PEG usage, whereas two studies reported an associative decrease in survival duration with PEG usage. A statistical meta-analysis was performed that combined the reported literature PEG cohort study results with the present study’s results to assess the overall trend. The overall or aggregate trend is a net positive and statistically significant associative increase in ALS survival duration with PEG usage as shown in Table 2. The meta-analysis finds that PEG users have a significant (*p* < 0.05) increase in survival duration (Figure 6) irrespective of ALS onset type. Note that for consistency, this meta-analysis used the “traditional” survival duration (time elapsed since patient-reported memory of first symptom until recorded date of death).

## 4. Discussion

The results of the present study and the overall meta-analysis indicate that ALS patient PEG usage has an associative survival benefit on survival duration. PEG usage is associated with an increase in survival duration of ALS patients regardless of limb or bulbar onset type (Figure 1). The timing (e.g., trimester or quartile) of PEG placement (Figure 3), alone, was not a predictor of associative benefit or functional outcome. However, FVC %predict at PEG placement was a significant predictor of associative benefit; patients with FVC %predict ≥ 50 at initial PEG placement fared significantly better, but those with FVC ≥ 60 at initial PEG placement saw the largest associative survival benefit (Figure 2). PEG usage significantly slowed weight loss and BMI decline. The present study was one of the first studies to show PEG usage was not associated with a significant worsening of clinician observed exuded patient mood. While PEG usage was associated with an overall positive associative survival benefit, PEG usage did not slow the functional pathology of ALS as assessed using the standard ALSFRS-R survey total score (Figure 4) or the adjunctively assessed patient gait score. Table 3 summarizes the primary study results and as well as the meta-analysis aggregate result (Figure 6).

The present study was one of the first to assess patient mood with PEG usage. Prior literature identified that 44% of ALS patients experience depression, and up to 30% suffer from anxiety [34]. Studies suggest a lack of intervention pertaining to the emotional and psychological state of the patient contributes to the reports of anxiety and depression among ALS patients [34,35]. Thus, it is important that ALS palliative interventions not further exude a significantly greater negative impact on patient mood. The novel CIM measurement lessens patient or caretaker survey bias; however, CIM still has notable limitations, such as being based on patients’ willingness to verbally disclose information about their moods or the doctors’ subjective assessments of patients’ moods based on non-verbal body language. Nonetheless, analytical findings using CIM support the contention that PEG placement and usage does not appear to be associated with a significantly greater decline in patient mood. More specific socio-psychology research is needed to verify impact of PEG usage on ALS patient mood and quality of life, preferably by constructing a method that can confidently quantify and balance impact of clinician impression, patient survey bias, and caretaker survey bias.

PEG usage did not slow the overall disease progression of ALS, as shown by the lack of significant difference in ALSFRS-R decline in PEG users versus non-users (Figure 4). However, only one question on the ALSFRS-R pertains to swallowing; most questions reference daily activities or breathing, which are only indirectly affected by caloric intake [16]. In short, PEG has a notable associative impact on survival duration but does not correlate with significant changes in functional metrics of ALS progression, albeit standardized ALSFRS-R scores or the novel visualized patient gait scores. These results indicate PEG is not directly impacting or slowing the disease etiology but rather providing a supportive means to increase survival duration, possibly through weight stabilization [23] or prevention of choking and/or dehydration. In short, PEG usage likely helps to comparatively maintain better overall health, which could help stave off the life-threatening infections, which are a key contributor to primary cause of death [29]. These results further support the great complexity of factors that correlate with or help to predict ALS patient survival [23,26].

The present study provided one of the largest sample sizes of ALS PEG users (Table 3), which assists in providing statistical power. However, it is important to assess the disparities of other ALS PEG studies in order to put results into context and to determine an overall trend, which can provide confident, real-world evidence for clinical decision support of PEG in ALS. There were discrepancies in observed results of prior published cohort studies included as part of the performed meta-analysis (Table 2): Some studies suggest an associative increase in survival with PEG usage [5,7,29,32,33], while others did not [30,31], and some studies report that PEG usage increases survival duration in bulbar onset patients but not limb onset patients [5]. Table 2 illustrates the findings of each study while listing the sample size, users and non-users. Sample size is believed to be a large contributor to the inconsistencies in the different results as most papers performed the studies on samples of 20–200 individuals. With larger and more diverse samples, results between studies could be more consistent to the true consensus. 

Beyond the present study, three prior published studies from the meta-analysis reported a statistically significant increase in disease duration as a result of PEG usage: Limousin, 2010 [7]; Spataro, 2011 [5]; and Burkhardt, 2017 [29]; with sample sizes of 63, 150, and 71, respectively. Another two studies reported an insignificant p-value but a qualitative increase in survival duration with PEG usage [32,33]. These 5 studies, along with the present study (for a total of 6 studies), were used to calculate an aggregate affect size, which is shown in Figure 6. This meta-analysis illustrates that there is a prominent and statistically significant increase in survival duration with PEG usage. Except for one study [1], the increase in survival duration was seen regardless of ALS onset type. 

In contrast, ALS PEG studies conducted by Strong, 1999 [31] and Chiò, 2004 [30], with sample sizes of 366 and 50, respectively, report a significant decrease in disease duration. However, there may be reasons other than PEG usage, alone, that explain why these two papers found unfavorable results. Chiò, 2004 [30] reported broadened patient inclusion criteria, including “probable ALS”. Strong, 1999 [31] examines gastrojejunostomy, which while similar to PEG, also has some key procedural and anatomical differences, which could impact analyzed outcome.

Overall results of the present study and the combined meta-analysis show PEG is a beneficial palliative intervention to treat ALS dysphagia and related symptoms. Specifically, PEG has an overall positive associative benefit on survival duration. The primary results of this study suggest PEG usage is not further impairing quality of life as measured via visualized clinical impression of mood. A previous post-mortem study of ALS PEG usage found the leading cause of death in patients appeared to be bronchopneumonia [29] rather than gastrointestinal or other site infections or complications directly related to PEG usage. Thus, overall analytical evidence to date strongly supports the use of PEG as a palliative measure for ALS patient care.

As previously noted, it is difficult to pinpoint what exactly is causing the associative increase in survival duration with PEG usage beyond the obvious impact on nutrition and hydration. Prediction of ALS survival is very complex, and current metrics do not fully convey the complexity and variance seen in heterogeneous ALS populations [23]. Antecedent disease and other patient medical history, lifestyle, or environmental exposure can also impact response to supportive care and especially overall survival duration [21,22,26]. Recent work of supportive non-invasive ventilation (NIV) also showed associative survival benefits that rivaled that of etiology-based pharmaceutical like Riluzole [26]. Many patients who use PEG also use NIV, especially at night [26,29]. It is not known for certain whether there is an additive or synergistic effect to using these two (or more) supportive interventions in combination. Recent ALS transgenic mouse work showed that oxidative stress therapeutics could also have functional impacts on quality of life in ALS by prolonging muscle function, even if impact on survival duration is minimal [36]. Thus, while we await a true etiological cure for ALS, palliative and supportive interventions like PEG provide an associative means to extend survival duration and improve quality of life for those patients currently battling this devastating disease.

## 5. Conclusions

PEG usage by ALS patients is associated with an overall significant increase in survival duration that is present regardless of ALS onset type. The primary results of this study confirm the current clinical standards put forth by the American Academy of Neurology, which recommends patients to have an FVC %predict ≥50 prior to prescribing PEG [28]. However, this study’s results do suggest that PEG placement while FVC %predict ≥60 is best for optimizing associative benefit. While PEG does not slow functional ALS pathology (ALSFRS-R), PEG usage also does not further harm quality of life as measured by the novel clinical impression of mood (CIM) metric. In summary, overall survival duration and quality of life evidence strongly supports PEG as a palliative intervention for ALS patients with dysphagia, weight loss nearing 10% of premorbid weight, and for patients who still have sufficient respiratory capacity at the time of PEG placement (e.g., FVC %predict ≥ 50).

## Figures and Tables

**Table 1:** Classification of included Amyotrophic Lateral Sclerosis (ALS) patients by percutaneous endoscopic gastrostomy (PEG) usage and ALS onset type.

ONSET TYPE	PEG User	PEG Non-User
Limb	138	448
Bulbar	133	164
Unclassifiable	7	37
Total	278	649

**Table 2:** List of PEG studies included in a meta-analysis to assess the overall associative impact of PEG usage with ALS survival duration. Prior cohort study results were combined with the present study to determine the aggregate associative effect of PEG usage on ALS patient survival duration.

PEG Study	Sample Size	PEG Users	Non-Users	*p* Value	Disease Duration with PEG
Limousin, 2010 [7]	63	33	30	*p* = 0.02	Significant Increase
Burkhardt, 2017 [29]	71	46	25	*p* < 0.01	
Spataro, 2011 [5]	150	76	74	*p* = 0.05	
Current study Bond, 2019	927	278	649	*p* = 0.0001	
Chiò, 2004 [30]	50	25	25	*p* = 0.004	Significant Decrease
Strong, 1999 [31]	366	73	293	*p* = 0.001	
Mathus-Vliegan, 1994 [32]	68	13	10	*p* = 0.6544	Insignificant Increase
Forbes, 2004 [33]	1226	142	1084	*p* = 0.52	
Aggregate Meta-Analysis	1578	352	1226	*p* = 0.0056	Significant Increase

**Table 3:** Summary of primary study results and the meta-analysis.

Parameter	Classification	Significant	*p*-Value
Disease Duration of PEG users vs. non-users	Limb Onset Bulbar Onset	Yes Yes	<<0.001 <<0.001
Disease Duration of PEG users	Limb vs. bulbar onset	Yes	<<0.001
ALSFRS-R decline in PEG Users vs. non-users		No	>0.05
Gait Score for PEG users	By PEG Placement Quartile	No	>0.05
Body Mass Index (BMI) in PEG users	Prior to PEG Following PEG	Yes No	<0.05 >0.05
Weight Change in PEG users	Prior to PEG Following PEG	Yes No	<0.05 >0.05
Time of PEG Placement	By Placement Trimester By Placement Quartile	NoNo	>0.05 >0.05
Clinical Impression of Mood (CIM) for PEG Users	By PEG Placement Quartile	No	>0.05
FVC %predict for PEG users at PEG placement	60% vs. <50% ≥50% vs <50%	Yes Yes	<0.05 <0.001
Meta-Analysis Assessing Associative Benefit of PEG usage on ALS Patient Survival		Yes	<0.05

## Supplementary Materials

The following are available online at https://www.mdpi.com/2076-3425/9/9/223/s1, Figure S1: Effect of PEG placement on clinical impression of mood (CIM) change. PEG users were divided into groups based on the relative disease quartile when PEG was placed: 1st Fourth (*n* = 61), 2nd Fourth (*n* = 50), 3rd Fourth (*n* = 49), and 4th Fourth (*n* = 27). The groups were then compared on the basis of overall change in CIM (*p* > 0.05), Figure S2: Effect of PEG placement on gait score decline. PEG users were divided into groups based on the relative quartile of disease duration at which PEG was placed: 1st Fourth (*n* = 61), 2nd Fourth (*n* = 50), 3rd Fourth (*n* = 49), and 4th Fourth (*n* = 27). PEG users and non-users were then compared on the basis of overall decline in gait score (*p* > 0.05). Results illustrate PEG usage has no impact on patient gait, Figure S3: Comparison of changes in body mass index (BMI) and body weight for patients in subpopulations categorized by quartile of initial PEG placement. (a) BMI change measured from the initial/first visit to the date of PEG placement (*p* < 0.05). (b) BMI change measured from the date of PEG placement to the last ALS clinic where weight was recorded (*p* > 0.05). (c) Change in patient weight measured from the initial/first visit to the date of PEG placement (*p* < 0.05). (d) Change in patient weight measured from the date of PEG placement to the last ALS clinic visit where weight was recorded (*p* > 0.05), Table S1: Keywords used in clinical impression of mood (CIM) scoring. A score of 1 indicates a negative mood, and a score of 0 indicates a positive or neutral mood, Table S2: Gait scoring categorization. A higher gait score indicates less ambulatory ability.
