# Supplementary material for: A Comprehensive Examination of Percutaneous Endoscopic Gastrostomy and Its Association with Amyotrophic Lateral Sclerosis Patient Outcomes
Source: Brain Sci. 2019 Sep 4;9(9):223. doi: 10.3390/brainsci9090223 (PMC6770872; doi:10.3390/brainsci9090223)
Supplement: Supplementary file 1 [file brainsci-09-00223-s001.pdf]

Supplementary Material: A Comprehensive Examination of Percutaneous Endoscopic Gastrostomy and Its Association with Amyotrophic Lateral Sclerosis Patient Outcomes

Leila Bond [1], Paulamy Ganguly[1], Nishad Khamankar[1], Nolan Mallet[1], Gloria Bowen[1], Braden Green[1], Cassie S. Mitchell[1*]

Supplementary Methods: Text mining for gait and mood scores

As discussed in the main manuscript Methods, two new scores (clinical impression of mood, CIM) and patient gait score were utilized as adjunctive measures to assess outcomes of PEG. Text mining was used to match clinician-utilized descriptions for each patient visit date in the electronic medical records and applied against a categorical scoring system. Because the patients were all from the same ALS clinic, there was overall consistent usage of language to enable this analysis.

Supplementary Table S1. *Keywords used in Clinical Impression of Mood (CIM) scoring.* A score of 1 indicates a negative mood, and a score of 0 indicates a positive or neutral mood.

Keyword	CIM Score
Anxious	1
Appropriate	0
Cheerful	0
Depressed	1
Dysthymic	1
Euthymic	0
Fair	0
Frustrated	1
Good	0
Irritable	1
Normal	0
Not good	1
Pleasant	0
Sad	1
Struggling	1

Supplementary Table S2. *Gait scoring categorization.* A higher gait score indicates less ambulatory ability.

Gait Category	Category Number
Independent	1
May use assistive device but appears steady	2
Partial gait (unsteady but able to stand and pivot)	3
Prone to falls (occasional or frequent)	4
Foot Drop/Steppage	5
Spastic	6
Altered arm swing or wide/narrow-based gait	7
Unable to walk at all	8

Supplementary Results: Impact of Clinical Impression of Mood

As noted in the Results of the main manuscript, there was no significant impact of PEG usage on clinical impression of mood when compared to PEG non-users in the same disease duration quartiles.

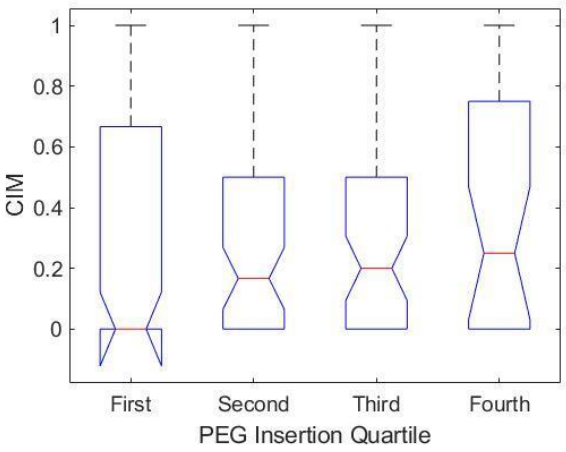

Supplementary Figure S1: Effect of PEG placement on Clinical Impression of Mood (CIM) change. PEG users were divided into groups based on the relative disease quartile when PEG was placed: 1st Fourth (n=61), 2nd Fourth (n=50), 3rd Fourth (n=49), and 4th Fourth (n=27). The groups were then compared on the basis of overall change in CIM ($p > 0.05$).

Supplementary results: Impact of PEG on gait score

Besides the standard ALSFRS-R score, we assessed an auxiliary metric of functional ALS decline--visualized patient gait score. The visualized patient gait score is based on clinician visualization of gait and not the patient's perception of their gait. Thus, it provides an adjunctive and potentially more agnostic means of assessing patient mobility. The median change in gait score decline between PEG users and non-users was 0 for every quartile with IQR ranging from 0.001-0.002 as shown in Supplementary Figure 2. Thus, for each disease quartile, there was no difference in median gait scores between PEG users and non-users. This result implies PEG usage has no impact on patient gait.

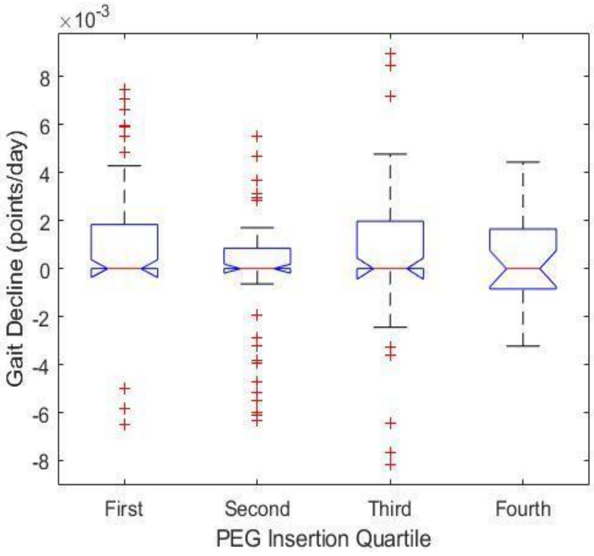

Supplementary Figure S2: Effect of PEG placement on gait score decline. PEG users were divided into groups based on the relative quartile of disease duration at which PEG was placed: 1st Fourth (n=61), 2nd Fourth (n=50), 3rd Fourth (n=49), and 4th Fourth (n=27). PEG users and non-users were then compared on the basis of overall decline in gait score ($p > 0.05$). Results illustrate PEG usage has no impact on patient gait.

Supplementary Results: Impact of PEG on BMI or Weight Change over ALS progression

As expected, PEG users' percent body weight decline and body mass index (BMI) decline from first ALS clinic visit until PEG placement was significant ($p < 0.05$). However, the percent body weight and BMI decline from PEG placement until last recorded ALS clinic visit was not significant ($p > 0.05$), as shown in Supplementary Figure 3.

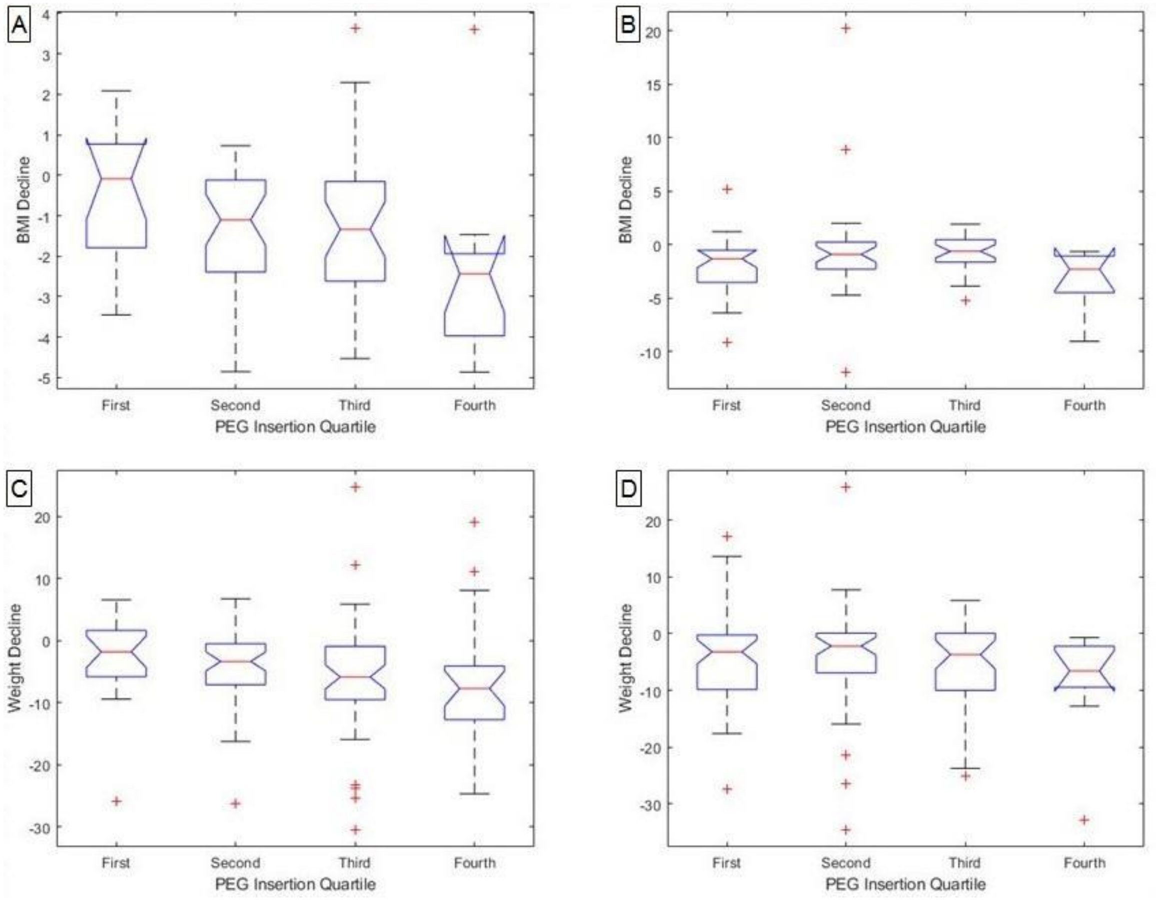

Supplementary Figure S3: Comparison of changes in Body Max Index (BMI) and body weight for patients in subpopulations categorized by quartile of initial PEG placement. **(a)** BMI change measured from the initial/first visit to the date of PEG placement ($p < 0.05$). **(b)** BMI change measured from the date of PEG placement to the last ALS clinic where weight was recorded ($p > 0.05$). **(c)** Change in patient weight measured from the initial/first visit to the date of PEG placement ($p < 0.05$). **(d)** Change in patient weight measured from the date of PEG placement to the last ALS clinic visit where weight was recorded ($p > 0.05$).
